# Improving Prediction Accuracy and Extraction Precision of Frequency Shift from Low-SNR Brillouin Gain Spectra in Distributed Structural Health Monitoring

**DOI:** 10.3390/s22072677

**Published:** 2022-03-31

**Authors:** Nur Dalilla Nordin, Fairuz Abdullah, Mohd Saiful Dzulkefly Zan, Ahmad Ashrif A Bakar, Anton I. Krivosheev, Fedor L. Barkov, Yuri A. Konstantinov

**Affiliations:** 1 School of Engineering and Physical Sciences, Heriot-Watt University Malaysia, Putrajaya 62200, Malaysia; n.nordin@hw.ac.uk; 2 Institute of Power Engineering, Universiti Tenaga Nasional, Kajang 43000, Malaysia; fairuz@uniten.edu.my; 3 Department of Electrical, Electronic & Systems Engineering, Faculty of Engineering & Built Environment, Universiti Kebangsaan Malaysia (UKM), Bangi 43600, Malaysia; saifuldzul@ukm.edu.my (M.S.D.Z.); ashrif@ukm.edu.my (A.A.A.B.); 4 Perm Federal Research Center of the Ural Branch of the Russian Academy of Sciences (PFRC UB RAS), 13a, Lenin Street, 614990 Perm, Russia; antokri@yandex.ru (A.I.K.); fbarkov@pstu.ru (F.L.B.)

**Keywords:** Brillouin scattering, distributed fibre-optic sensors, data processing, machine learning, BFS extraction, BOTDA, concrete, structural health monitoring

## Abstract

In this paper, we studied the possibility of increasing the Brillouin frequency shift (BFS) detection accuracy in distributed fibre-optic sensors by the separate and joint use of different algorithms for finding the spectral maximum: Lorentzian curve fitting (LCF, including the Levenberg–Marquardt (LM) method), the backward correlation technique (BWC) and a machine learning algorithm, the generalized linear model (GLM). The study was carried out on real spectra subjected to the subsequent addition of extreme digital noise. The precision and accuracy of the LM and BWC methods were studied by varying the signal-to-noise ratios (SNRs) and by incorporating the GLM method into the processing steps. It was found that the use of methods in sequence gives a gain in the accuracy of determining the sensor temperature from tenths to several degrees Celsius (or MHz in BFS scale), which is manifested for signal-to-noise ratios within 0 to 20 dB. We have found out that the double processing (BWC + GLM) is more effective for positive SNR values (in dB): it gives a gain in BFS measurement precision near 0.4 °C (428 kHz or 9.3 με); for BWC + GLM, the difference of precisions between single and double processing for SNRs below 2.6 dB is about 1.5 °C (1.6 MHz or 35 με). In this case, double processing is more effective for all SNRs. The described technique’s potential application in structural health monitoring (SHM) of concrete objects and different areas in metrology and sensing were also discussed.

## 1. Introduction

The most used material in engineering structures is concrete. The list of its varieties is constantly expanding [1,2]. Mechanical phenomena, both during concrete hardening and during operation [3] and ageing [4] of particularly critical concrete structures, such as hydraulic structures, are important data that require analysis and study. This is the scientific direction of structural health monitoring (SHM) [5], which is actively developing due to the emergence of new materials [6]. The challenging issue that SHM helps to solve are related to the ability to receive data directly from construction in real-time about their mechanical state during all its operational life. This allows the critical defects in the structure to be found in time, thus preventing disasters. The most used instruments in SHM are point-wise [3] and distributed fibre-optic sensors (DOFS) [7,8,9]. DOFS are mostly based on various principles of optical reflectometry. Most often, using such techniques, the spatial distribution of temperature and deformation is studied. There are a number of applications where obtaining accurate data on the temperatures and strains of a material is essential. For example, in [10] the authors illustrate the age-induced degradation on the Rayleigh-based distributed sensor response. To implement such a task, the distributed systems must have a resolution of the order of tenths of a microstrain. In the case of using a system based on the Brillouin scattering principle, in terms of Brillouin frequencies, this will be in units of kilohertz [11]. When using experimental materials and designs, optical fibres can be squeezed, pinched, or stretched more than they originally intended to. In such cases, the optical signal undergoes significant changes—it is distorted causing the signal-to-noise ratio to dramatically decreases [12,13,14]. At the same time, to solve a scientific or industrial problem, sensors still need to give as correct values as possible. In the case of the aforementioned sensor systems based on Rayleigh scattering, these issues are not so acute since this type of scattering is relatively strong in comparison to Brillouin scattering which is widely used in DOFS [15,16]. Meanwhile, systems based on these principles are applied in SHM quite often [17,18]. One of the serious problems (especially at low signal-to-noise ratios) is the inability to accurately determine the frequency maximum of the Brillouin gain spectrum [19]. The present work is focused on solving this problem using a combination of various methods, including machine learning. Since the available equipment allowed us to train the system only on thermocouple readings, all issues of determining the frequency shift will be solved using degrees Celsius, which will later be converted to frequency and strain.

Thus, obtaining the correct BFS value is critical for the efficient operation of this type of distributed sensor. In conditions of extremely low signal-to-noise-ratio (SNR), when, for example, an optical line has a sufficiently high attenuation due to the use of a special type of optical fibre or external influence, this task becomes even more difficult. The methods known to the authors for revealing the real value of the maximum of the Brillouin gain spectrum (BGS) can be divided into four possible approaches:

1. The first group of methods requires retrofitting additional hardware or software-based digital filters to increase the SNR of BGS. For example, in [20,21,22], the low-pass filtering, pump and probe waves intensities modulation and also the modulation of the probe signal wavelength were used. The reports in [23,24] describe the successful use of the wavelet transformations designed for signal filtering. The techniques mentioned above showed their effectiveness, but due to their complexity (the use of atypical and sophisticated algorithms with a significant number of parameters and coefficients) and sensitivity to the spectra shape, the implementation of these methods is limited. The idea of increasing the number of optical pulses for the strain measurement in a single repetition time has also been proposed. Inspired by the pulse coding technique in radar technology, the incorporation of optical pulse coding techniques such as Golay complementary codes has also been proposed for the purpose of improving the SNR [25].
2. Then, the most popular in commercial instruments and well-known method is the reconstruction of a Lorentzian function, which is the actual BGS shape. This method is widely used in engineering applications [26]. In recent years the method has seen significant improvements in BFS detection precision [27] as well as in calculation speed increase and works perfectly if the tuning coefficients of the Lorentzian shape are precisely extracted. However, in practice, due to digitization data losses and the noise contributed by various other reasons, the spectrum can be significantly distorted [28,29]. These problems bring new limitations to the BGS reconstructing method’s application.
3. The third approach is based on calculating the cross-correlation function of the obtained BGS and some previously generated profiles of the Lorentzian shape [30,31,32,33]. Additionally, instead of this approach, the spectrum can be obtained by inverting the original BGS, which is called backward correlation. This method will be described in this article in more detail later. Practice has shown that correlation methods are quite effective in studying signals with low SNR.
4. Another noteworthy method is machine learning [34,35,36,37]. These are artificial intelligence methods used to obtain the correct characteristic feature which is not a direct solution to the problem but is a learning input through applied solutions to many other similar problems. To obtain the desired value of the frequency shift, it is advisable to use learning from precedents, or inductive learning based on identifying empirical patterns in the data. Similar methods, including the generalized linear model (GLM) method, which will also be discussed in detail in this article, have become widespread and have demonstrated their effectiveness not only in increasing the accuracy of determining the BFS but also in predicting it.

**Figure 1:**
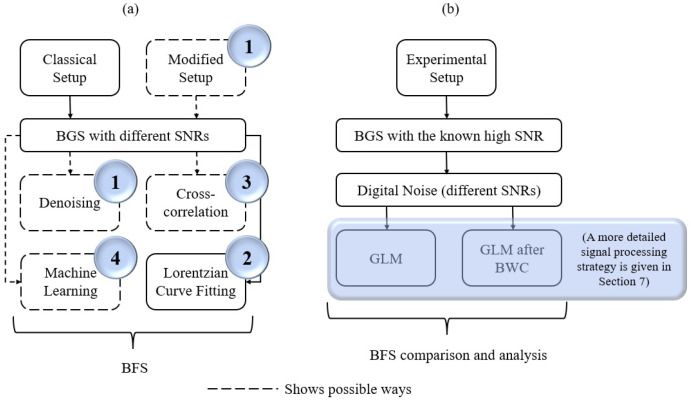
The classical approach of the BFS extraction process (**a**) in comparison with the proposed study (**b**).

All these ways of BFS extraction are presented in a simplified manner within the process of temperature or strain measurements using distributed Brillouin scattering technique (Figure 1a). The circled numbers correspond to the numbers of the approaches in the list above.

Since the GLM method has demonstrated its effectiveness exclusively on the raw BGS so far, its potential use for processing previously stored or newly received data that has already been processed by other methods becomes especially interesting. This work is focused on the consistent application of BFS extraction methods, and their effect on the detection precision and the prediction accuracy evaluated at different signal-to-noise ratios.

In the sections below, firstly, the theory of the methods used and the ability of their joint application and possible advantages of this approach are briefly discussed. Then the conducted experiments are described, providing the processing details of the obtained data and then demonstrating that the subsequent use of backward correlation method and generalized linear model (Figure 1b) help to improve the BFS prediction efficiency as well as measurement precision in a wide range of SNRs. The classical solutions which usually apply the single processing with one of the well-known methods show smaller efficiency. The Lorentzian curve ditting method realized with the Levenberg–Marquardt (LM) algorithm is one of them. Its theory basics are presented in Section 2.

## 2. Levenberg–Marquardt (LM) Theory

The most used method to obtain BFS is the Lorentzian curve fitting (LCF) as the measured BGS resembles a Lorentzian shaped curve [38]. Most of the LCF based methods require initialization of model parameters. The accuracy of the system, however, is solely dependent on the BFS determination [39,40]. This means that the pre-selection of the spectral range is crucial for the fitting to be precise. One method to realize the fitting technique is by using a Levenberg–Marquardt (LM) algorithm. The LM algorithm is a non-linear least square algorithm that has a good convergence property to minimize errors in the derived BGS spectra. In this paper, the LM algorithm implemented is from the *lmfit* module in Python. The module supports the optimization method and is advantageous when encountering curve fitting related problems, which as mentioned previously is mainly due to the initial parameter setting that could eventually affect the accuracy of the whole system. This *lmfit* module automatically calculates the structure factor, convoluted with the instrumental resolution, and then calculates the least square minimization to compute the optimal parameters with high precision.

The Lorentzian model in the *lmfit* module is based on a Lorentzian or Cauchy–Lorentz distribution function. The model possesses three main parameters: amplitude (*A*), width (*w*) and peak position (μ). The model is expressed as:

(1) $$f\left(x:A,\mu ,w\right)=\frac{A}{\pi }\left[\frac{w}{{\left(x-\mu \right)}^{2}+w^{2}}\right]$$

Subsequently, the FWHM can be translated as 2w. Whereas the established Brillouin unsaturated gain profile g(v) of stimulated Brillouin scattering is expressed as:

(2) $$g\left(v\right)=\frac{g_{B}}{1+{\left[\frac{\left(f-f_{B}\right)}{\frac{\Delta f_{B}}{2}}\right]}^{2}}$$

where $f_{B}$ is the central frequency (which in this study is the BFS) and $\Delta f_{B}$ is the BGS linewidth, and $g_{B}$ is the peak of the spectrum. The *lmfit* module possess a *model.guess* function that enables it to guess the starting values for the model parameters through an iterative algorithm. From this method, some values for data are taken and utilized to construct reasonable starting values for the parameters. As the set of parameters is obtained, the module will subsequently perform a curve fit onto the data array. These methods are repeated for each of the data arrays. Compared to the conventional LCF method, the LM based LCF using *lmfit* is much faster in terms of processing as it is automatically and relatively more accurate in guessing the starting values for the model parameters.

## 3. Backward Correlation (BWC) Method Theory

**Figure 2:**
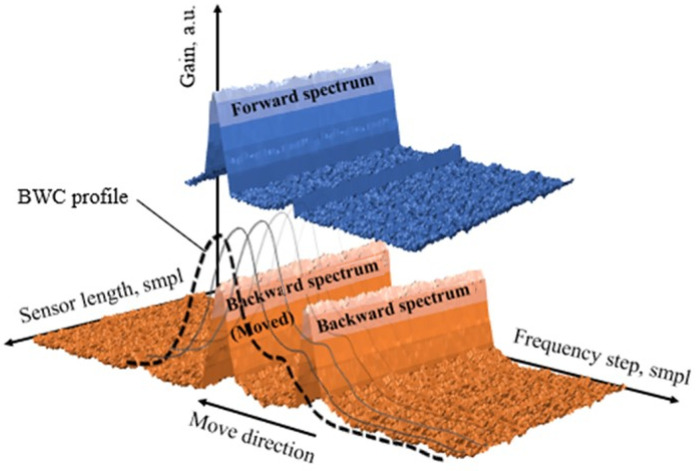
A schematic diagram combined using real data, describing the method operation principle (blue graph—forward spectrum; orange graph—backward spectrum with its shifted copy; dotted line—backward correlation function).

Correlation methods can be used as an alternative to the LCF/LM methods. It was previously shown [41] that the backward correlation (BWC) method, described in detail in [42], demonstrates good efficiency in finding the maximum of the spectrum in conditions of low signal-to-noise ratios, distortion of the spectrum shape and even digitization defects. In [41], extreme detection conditions were created using digital simulation. How effective this method will be in processing data obtained by a hybrid method—an experimental setup and subsequent regeneration of noise—is reflected in one of the parts of the current study. A basic theory of the BWC method is presented in Figure 2.

Let us imagine that we have registered a BGS using a typical Brillouin optical time-domain analysis (BOTDA) or Brillouin optical time-domain reflectometry (BOTDR) setup. After analogue-to-digital conversion, it can be written as a discrete sample set. This sample set could be presented as 2N+1 values of $P_{i}$ (the backscattering power at frequency $f_{i}=f_{0}+i*\Delta f$$\left[f_{0}+i*\Delta f,P_{i}\right]$), where *i* is in the range of [0; 2N] and corresponds to the current frequency in BGS obtained from measurement; $f_{0}$, the scanning start frequency; and Δf is a frequency sweeping step. You can see the array of $P_{i}$ values represented graphically in Figure 2, coloured blue. The obtained source data could be described as two components. The first one is the useful data itself, the second component is the noise, distorting the obtained signal: $P_{i}=P_{i}^{s}+P_{i}^{n}$. In the ideal case, when no noise is present, i.e., $P_{i}^{n}=0$, a simple iterative spectral peak seeking could be used to extract the precise BFS value up to analogue-to-digital conversion error. However, in [42] we have previously demonstrated that even the insignificant noise (SNR less than 20 dB) leads to precision loss. Let us write down the source data $P_{i}$ back to front to get the “backward” BGS $P^{\prime }i=P_{2N-i}$. To calculate the correlation function we need also to provide the “shift” *k* between two these data arrays, so the backward signal with the shift *k* could be presented as $P^{\prime \prime }i=P_{i-k}^{\prime }$, considering that $P^{\prime \prime }=0$ in the case when j=[i−k] is out of [0;2N] range. The parameter *k* is an integer in the range of [−2N;2N]. Let us present the resulting signal as a convolution of the forward and the backward signals:

(3) $$X=\sum_{i=0}^{2N}P_{i}*P_{i}^{\prime \prime }=\sum_{i=0}^{2N}P_{i}^{s}*P_{i}^{{}^{\prime \prime }s}+\sum_{i=0}^{2N}P_{i}^{n}*P_{i}^{{}^{\prime \prime }s}+\sum_{i=0}^{2N}P_{i}^{s}*P_{i}^{{}^{\prime \prime }n}+\sum_{i=0}^{2N}P_{i}^{n}*P_{i}^{{}^{\prime \prime }n}$$

where ∗ means multiplication. It is clearly seen from Equation (3) that the second and third parts equal to zero, accurate to a statistical error since the noise and clean BGS data of the resulting signal are not related to each other. The similar situation with the fourth term which is also close to zero since the convolution of two independent noise signals gives extremely low values. The first term represents the clean signals convolution. The graphical interpretation of Equation (3) is a BWC profile (Figure 2, dotted line).

To have the right to apply other techniques after the BWC-profile calculation, we must estimate the curve shape at first [43]. The convolution of two Lorentzian functions shifted relative to each other by some value δ is given by (here we neglect discretization effects and transit from summation to integration)

(4) $$I\left(f\right)=\int_{-\infty }^{\infty }\frac{w^{2}\pi^{-2}}{\left[{\left(f-f_{b}\right)}^{2}+w^{2}\right]\left[{\left(f-f_{b}-\delta \right)}^{2}+w^{2}\right]}df$$

where *w* is the spectrum width and $f_{b}$ is the BFS. For simplicity, at this stage, let us set the $f_{b}$, equal zero. We come to:

(5) $$I\left(f\right)=\frac{w^{2}}{\pi^{2}}\int_{-\infty }^{\infty }\frac{df}{\left[f^{2}+w^{2}\right]\left[{\left(f-\delta \right)}^{2}+w^{2}\right]}=\frac{w^{2}}{\pi^{2}}\int_{-\infty }^{\infty }\frac{df}{\left[\left(f-z_{1}\right)\left(f-z_{2}\right)\right]\left[\left(f-z_{3}\right)\left(f-z_{4}\right)\right]}$$

Here $z_{1-2}=\pm i*w$, $z_{3-4}=\delta \pm i*w$ are the points of singularity where denominator turns to zero.

**Figure 3:**
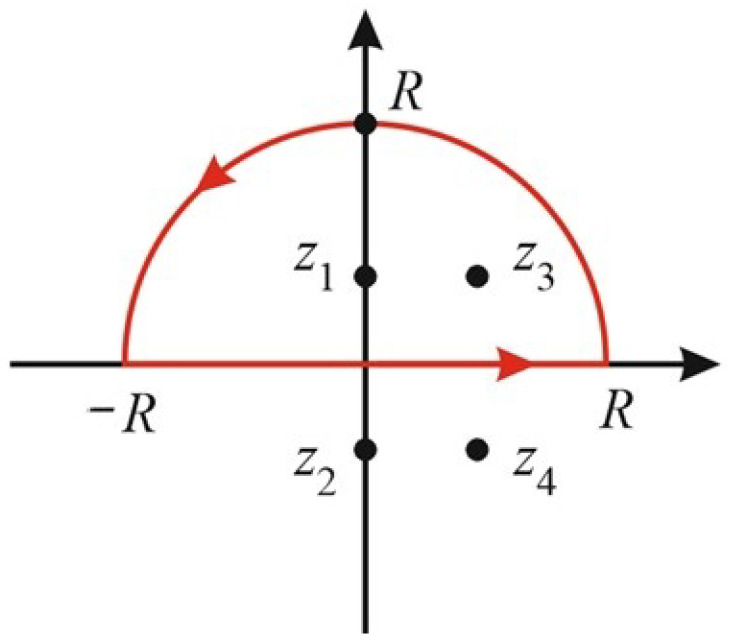
Illustration of taking the integral procedure.

Let us choose the closed curve consisting of section [−R,R] and semicircle in half-plane where the imaginary part is positive—as shown in Figure 3. According to residue theorem, the integral of the function under consideration over the closed curve is equal to 2πi∑resf and summation is taken over all the singularity points inside the curve—i.e., over $z_{1}$ and $z_{3}$. In the limit R→∞, the integral over the semicircle tends to zero (since the semicircle length is proportional only to *R* and the integer and falls as $1/R^{4}$). Obviously, the integral over the [−R,R] section tends to the required value *I*. Let us calculate the residues at points $z=z_{1}$ and $z=z_{3}$:

(6) $${Res\;f|}_{z=iw}=\frac{1}{\left(iw+iw\right)[{\left(iw-\delta \right)}^{2}+w^{2}]}=\frac{1}{2iw[\delta^{2}-2iw\delta ]}$$

(7) $${Res\;f|}_{z=\delta +iw}=\frac{1}{\left(\delta +iw-\delta +iw\right)[{\left(iw+\delta \right)}^{2}+w^{2}]}=\frac{1}{2iw[\delta^{2}+2iw\delta ]}$$

Finally, we can write

(8) $$\begin{array}{c} I=2i\frac{w^{2}}{\pi }\left[\frac{1}{2iw[-2iw\delta +\delta^{2}]}+\frac{1}{2iw[2iw\delta +\delta^{2}]}\right]=\\ \frac{w}{\pi }\left[\frac{2\delta^{2}}{\left[2iw\delta -\delta^{2}\right]\left[2iw\delta +\delta^{2}\right]}\right]=\frac{\left(2w\right)}{\pi }\left[\frac{1}{\left[{\left(2w\right)}^{2}+\delta^{2}\right]}\right] \end{array}$$

(9) $$I\left(\delta \right)=\frac{\left(2w\right)}{\pi }\left[\frac{1}{\left[{\left(2w\right)}^{2}+\delta^{2}\right]}\right]=\frac{\left(2w\right)}{\pi }\left[\frac{1}{\left[W^{2}+\delta^{2}\right]}\right]$$

where *w* is a FWHM of the BWC profile.

It is obvious that expression Equation (9) is also a Lorentzian function, and, therefore, has a clearly localized maximum, associated with the desired value. As mentioned above, the Lorentzian-like shaped signal makes it possible to include the method into processing circuits. The value of “optimal shift” δ is fairly easy to be recalculated in BFS.

## 4. Generalize Liner Model (GLM) Theory

**Figure 4:**
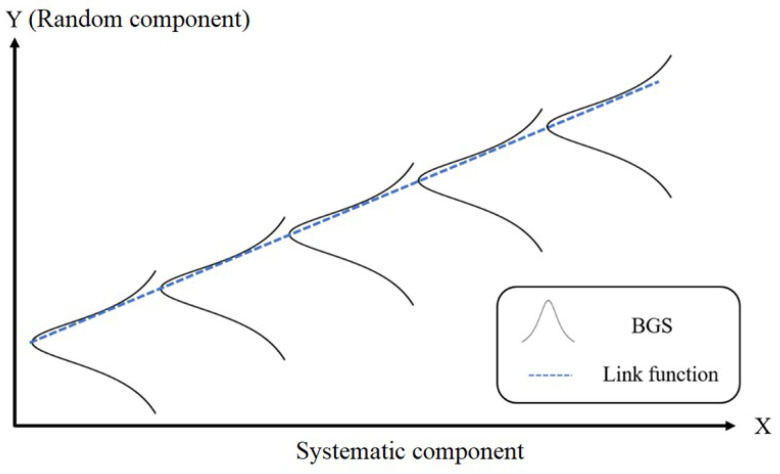
The use of GLM for BFS extraction.

GLM is an extension of the general linear model in machine learning that could compute response based on the maximum likelihood of the training set of data [44,45]. It allows Lorentzian or Gaussian, Poisson, normal and a few other distribution-like data to be processed through GLM. The model calculates the mean and matches it to the linear predictor using a link function with reweighted least square iterative technique onto the distribution data to produce the maximum likelihood predictions. There are three main components in GLM: the random component, systematic component and link function. The link function, as its name suggests, links the other two components together. The random component, which is the distribution data, with the linear predictor of the systematic component is given by:

(10) $$\eta_{i}=\beta_{0}+\beta_{1}X_{i1}+\beta_{2}X_{i2}+\ldots +\beta_{k}X_{ik}$$

where $\beta_{k}$ are the estimated coefficients, and $X_{ik}$ are the independent variables. Then, a commonly employed link function or identity link transforms the expectation of the dependent variable $\mu_{i}\equiv E\left(Y_{i}\right)$, to the linear predictor, which is given by:

(11) $$g\left(\mu_{i}\right)=\eta_{i}=\beta_{0}+\beta_{1}X_{i1}+\beta_{2}X_{i2}+\ldots +\beta_{k}X_{ik}$$

where $\mu_{i}$ is the expected value of the response. GLM can also be thought of as a linear model for a transformation of the expected response. The physical model can be represented as in Figure 4. Since the behaviour of BFS is linear to the increment of temperature, the GLM suits the BFS–temperature input–output target for the BOTDA dataset the best.

The random component, which is represented as *Y* value, is the BGS distribution, and the systematic component is represented as *X*. For instance, in this paper, the temperature categories for each BGS, represented by $Y_{1},Y_{2},\ldots ,Y_{n}$, are all independently distributed. The task of the link function is to connect the two variables together.

Before implementing the GLM model, there are a few crucial steps required. First, the BGS distribution along the fibre was retrieved experimentally using the experimental setup presented below. The BGS distribution is normalized to 1 in order to increase consistency and easier object to target mapping. Next, the target value, which in this case is the temperature measured by the thermocouple, was paired with the noisy raw BGS. The BGS–temperature pairs will create a dataset that will be used in the training phase in GLM model.

In the training phase for machine learning models, it is crucial to avoid overfitting. Overfitting can eventually lead to low accuracy and has a higher chance to occur when the dataset used is noisy. A noisy dataset may not represent the actual properties of that particular dataset. Forcing the model to learn such a noisy dataset may make the model more flexible but, at the same time, can cause overfitting. However, for a different type of machine learning model, a different technique or solution is required to overcome this and, at the same time, increase the interpretability of the model.

For GLM, the technique used is called regularization. Regularization tunes the learning algorithm from relying too much on every data point and helps identify the significant predictors. It reduces the variance of the model significantly without a substantial increase in its bias. The regularization technique used in this paper is called Lasso, which stands for the least absolute shrinkage and selection operator. The Lasso regularization can be expressed as:

(12) $$\sum_{i=1}^{n}{\left(y_{i}-\beta_{0}-\sum_{j=1}^{p}\beta_{j}x_{ij}\right)}^{2}+\lambda \sum_{j=1}^{p}\left|\beta_{j}\right|=\mathrm{RSS}+\lambda \sum_{j=1}^{p}\left|\beta_{j}\right|$$

where the first term is residual sum of squares (RSS) and β represents the coefficient responsible for estimating different variables. The Lasso method allows variable selection that can yield sparse models. This means that Lasso shrinks the insignificant features or completely removes some features altogether in order to avoid over-fitting.

## 5. Experiment Setup

**Figure 5:**
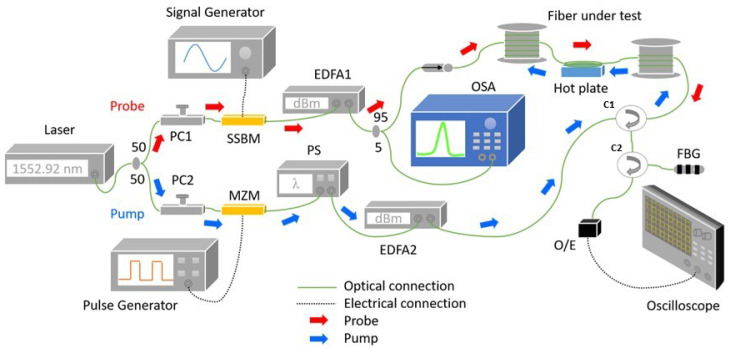
Experimental BOTDA setup.

The experimental setup for BOTDA is illustrated by Figure 5. A continuous-wave (CW) laser source having a linewidth of 100 kHz was used as an input signal with a centre wavelength at 1552.92 nm. The source radiation was split by a 50:50 optical coupler into a CW probe and pulsed pump signal. The probe arm is comprised of three main components: a signal generator, a single side-band modulator and an erbium-doped fibre amplifier. The CW light was modulated with a high-frequency radio signal by the SSBM which generates a carrier suppressed double-sideband probe signal. The radio frequency was swept at 1 MHz scanning step between 10.765 to 10.935 GHz, which is the typical range of BGS for a single-mode fibre. The modulated signal was amplified to 0 dBm by EDFA1 and then sent into the fibre under test (FUT). The FUT was subjected to five different temperature levels, 45 °C to 85 °C with 10 °C increment.

The pump arm consists of a pulse generator, Mach–Zehnder modulator, polarization scrambler and another amplifier. The light was modulated with a square pulse by MZM to generate the pump signal with an extinction ratio of around 25 dB. The polarization of this pump signal was scrambled by the PS to reduce the polarization-dependent noise in the Brillouin signal. The power of this pump was boosted by EDFA2 and launched into the opposite end of the FUT.

The Brillouin signal from FUT was passed through a fibre Bragg grating (FBG) to filter out other spectral components such as the Rayleigh scattering and the anti-Stokes frequency. The signal was converted into electrical by a DC-coupled 1 GHz bandwidth detector and digitized by an oscilloscope with 500 MHz bandwidth, 2.5 Gbps sampling rate and averaged 5000 times. For this particular experiment, the FUT was a 1.26 km long standard telecom-grade single-mode fibre. A short section of the FUT, about 8 m, was heated to the set temperature using a hot plate. The thermocouple was used to measure the temperature throughout the experiment as a reference. Table 1 lists the make and model of key components and devices used in the experiment.

## 6. Digital Noise

Further, the noise had to be added to the obtained spectra. This could be done in several ways: for example, to introduce additional attenuation of the optical signal into the line by adding macro bends or other defects; controllably shift the area of interest to the end of the line; reduce the duration of the probe pulses; the addition of digital noise. Using the first two listed methods, it is rather difficult to achieve noise control with good accuracy, the third method can distort the useful signal form, which will cast doubt on the correctness of the experiment. The fourth way, associated with the generation of digital noise based on spectra previously obtained in the experiment, is preferable in view of its simplicity, transparency and lack of influence on other parameters of the study.

However, digital noise must be generated correctly. It is possible to study the distribution law of noise intensities in the experimentally obtained spectra and generate new noise according to this law. This approach is appropriate if we assume the linearity of the photodetector response over the entire frequency range of interest. If we assume that the response, and therefore the noise characteristics at each individual frequency, can vary, it is necessary to create noise with this feature in mind. To do this, we took a big set of individual spectra, exclude the useful signal from them, and, based on the data obtained, generate noise for each frequency separately. Let us denote these BGS sets, obtained for each sensor temperature, by the matrix *N*, in which *I* is the signal intensity at the discrete value of the frequency *m*.

(13) $$N=\left(\begin{array}{ccc} I_{1;1} & \cdots & I_{m;1}\\ \vdots & \ddots & \vdots \\ I_{1;n} & \cdots & I_{m;n} \end{array}\right)$$

Thus, *m* is the number of elements in the spectrum; *n* is the number of spectra for a given temperature. The values of the matrix are calculated from the matrix *N*:

(14) $$B=\left(\begin{array}{ccc} n^{-1}\sum_{i=0}^{n}N\left(I_{1;i}\right) & \cdots & n^{-1}\sum_{i=0}^{n}N\left(I_{m;i}\right)\\ \vdots & \ddots & \vdots \\ n^{-1}\sum_{i=0}^{n}N\left(I_{1;n}\right) & \cdots & n^{-1}\sum_{i=0}^{n}N\left(I_{m;i}\right) \end{array}\right)$$

It is easy to see that the rows of this matrix represent the averaged BGS for each individual temperature, and therefore for a certain BFS. Further, the set of noise components $N^{\prime }$ was calculated by subtracting the matrix *B* from the matrix *N*:

(15) $$N^{\prime }=N-B$$

Thus, the columns in this matrix will contain deviations from the average spectrum inherent for every frequency of the spectrum. If for each discrete frequency *m* we randomly choose the number of the element in the column [0,n], then the laws of the intensity distribution of the noise components for each frequency will be maintained.

The resulting noise must be added to the original spectrum with a known signal-to-noise ratio, multiplied by the appropriate factor, as shown in the equation below:

(16) $$S=10^{\frac{N(add)}{10}}(N^{\prime }\left(I_{1;n^{\prime }}\right)\ldots N^{\prime }\left(I_{m;n^{\prime }}\right)+\left(N\left(I_{1;n}\right)\ldots N\left(I_{m;n}\right)\right)$$

where N(add) is the specified increase in noise, dB; $n^{\prime }=\mathrm{random}\left(0\ldots n\right)$ for the column corresponding to the frequency *m*.

We believe that this approach will allow the most realistic reproduction of the BGS noise pattern. The next section considers the application of Equation (16) to generate digital noise with a given SNR.

## 7. Data Processing Strategy

**Figure 6:**
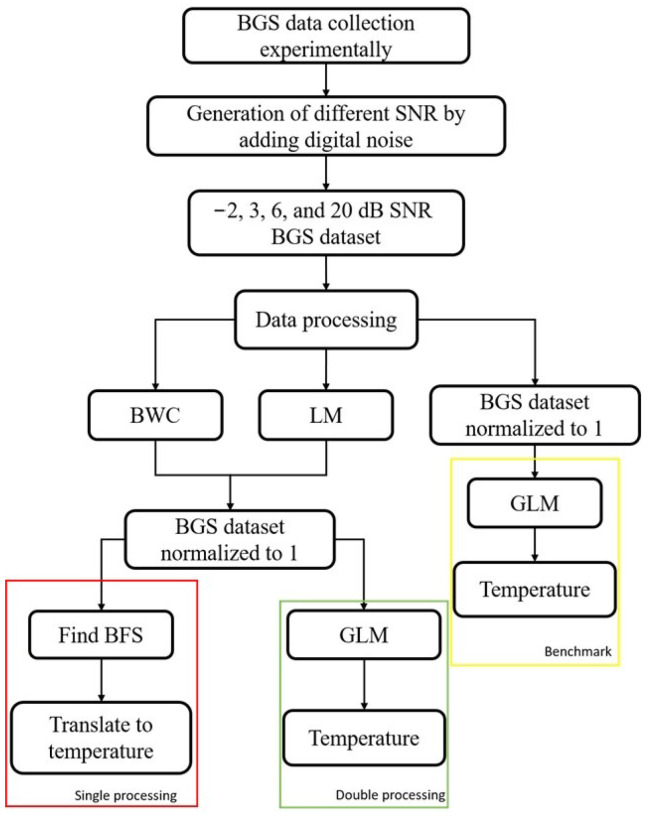
Data processing principle.

Once the data of five different temperature conditions have been collected experimentally, the BGS is distorted by digital noise as described in the previous section. There are four different values of SNR that had been generated through digital noise: −2, 3, 6 and 20 dB, comprising four datasets with five different temperature conditions in each of the datasets. The complete steps of the whole data processing areas are visualised in Figure 6. Once the added digital noise dataset is generated, the following step is processing the spectra with the LCF- and correlation-based methods: LM and BWC. The purpose of this step is to improve the noisy spectra by replacing them with a smooth Lorentzian curve. Next, the new spectra are normalized to unity for better efficiency when using the machine learning method in the later stage.

There are two ways of obtaining the temperature corresponding to the individual BGS. One way is to obtain the central frequency of the spectra, i.e., the BFS and the other one is by using the machine learning method. For the former method, the fibre coefficient has to be calculated through BFS–temperature slope and then utilizing it for the translation, as what had been done in single processing. The second method is by using a machine learning model. As previously mentioned in this paper, the machine learning model used is GLM. The GLM model is trained with the four datasets (−2, 3, 6 and 20 dB) containing 100 BGS of each of the temperature conditions and then tested with 100 spectra samples collected at 75° for double processing. Another GLM model is trained with the noisy spectra and then tested with the same 100 spectra samples collected at 75 °C as a benchmark. The described principle is shown in Figure 6.

## 8. Results and Discussion

**Figure 7:**
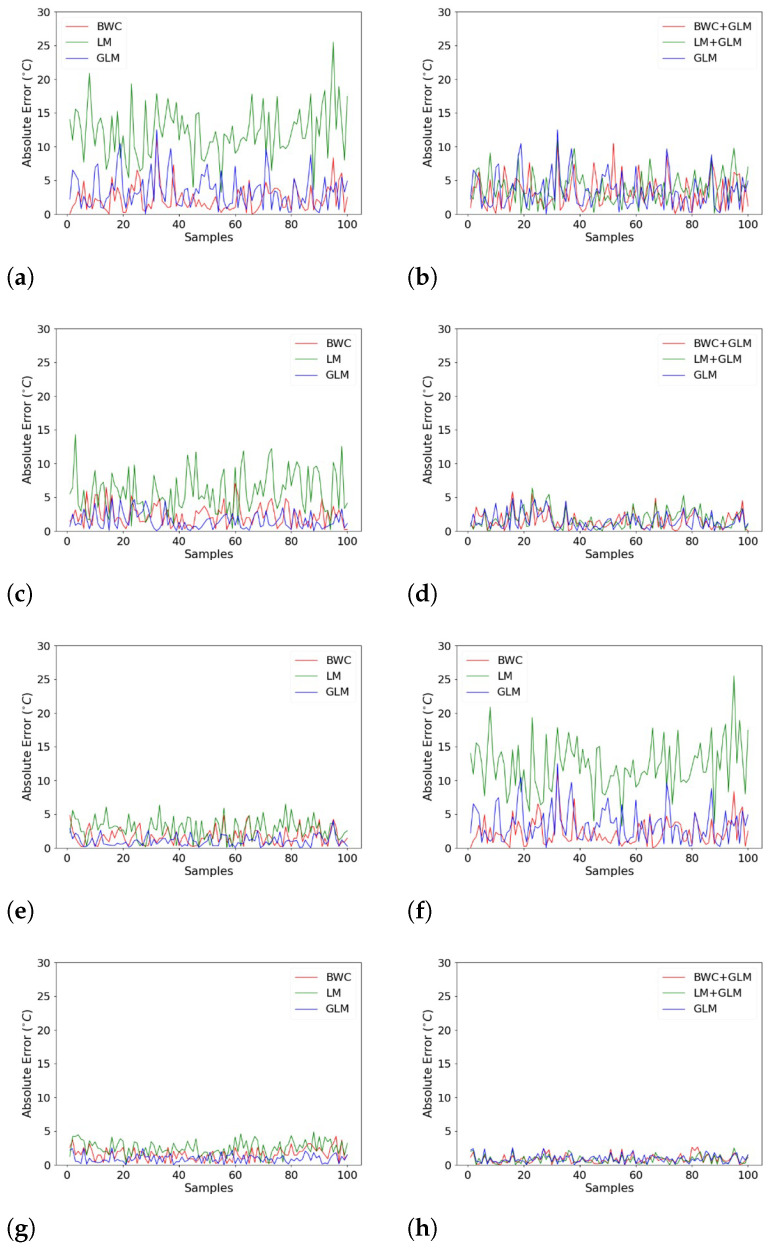
The absolute error results obtained after data processing: (**a**) −2 dB single processing; (**b**) −2 dB double processing (**c**) 3 dB single processing; (**d**) 3 dB double processing; (**e**) 6 dB single processing (**f**); 6 dB double processing; (**g**) 20 dB single processing; (**h**) 20 dB double processing.

To identify the efficacy of the proposed method, the 100 BGS spectra collected at 75 °C are plotted for each SNR condition. The absolute error results are as illustrated in Figure 7. The absolute error is calculated based on the difference between the thermocouple reading, where we regarded it as the true temperature of 75 °C and the temperature predicted by the GLM model and through the BFS calculation process. The x-axis represents the fibre length described by 100 spectra samples collected at 75 °C. On the other hand, the y-axis represents the absolute error calculated for each of the individual 100 BGS.

On the left-hand side, the graphs are plotted for single processing methods (BWC, LM), as opposed to the right-hand side where the BGS had been processed through double processing methods (BWC + GLM, LM + GLM). All the plots are inclusive of the benchmark plot where the noisy spectra were processed using only GLM. Visually, one can notice that the absolute errors were improved significantly using the double processing methods. Of all four of the SNR conditions, the single processing method appeared to have higher error as opposed to the benchmark. However, when going through the double processing method, the error is comparable, and some are even better than the benchmark.

For -a 2 dB SNR, the maximum error for single processing using LM is 25.5 °C whereas when using the double processing method (LM + GLM) it is 11 °C, an improvement of 14.5 °C. On the other hand, for single processing using BWC, the error is 11.2 °C and for the double processing method (BWC + GLM), it is 10.9 °C. The maximum error for benchmark, however, is 12.5 °C, higher than both double processing methods.

As for a 3 dB SNR, the difference in error between single and double processing for LM is 7.9 °C and 1.3 °C for BWC. While for 6 dB SNR, the error has improved by 0.9 °C for the double processing method using LM and 1.4 °C for the double processing method using BWC. Finally, for 20 dB SNR, the improvement using the double processing method is by 2.4 °C for LM and 1.7 °C for BWC.

**Figure 8:**
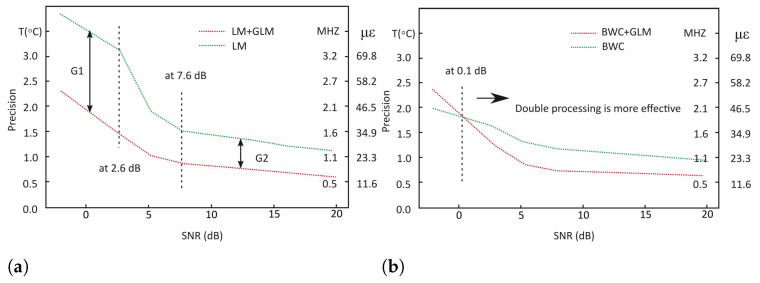
Measurement precision demonstrated by studied methods: (**a**) LM; (**b**) BWC.

**Figure 9:**
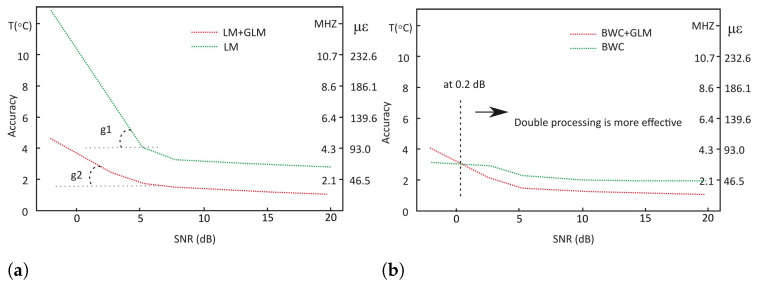
Prediction accuracy demonstrated by studied methods: (**a**) LM; (**b**) BWC.

For a deeper insight, the measurement precision and the prediction accuracy were calculated and shown in Figure 8 and Figure 9. The prediction accuracy is calculated based on the root mean square error (RMSE) formula taking the thermocouple reading, 75 °C, as the true temperature. On the other hand, the measurement precision is calculated based on the standard deviation. The temperature measurement precision represents the statistical variability and how reproducible measurements were even if the points are far away from the actual temperature reading. In contrast, the temperature prediction accuracy reflects on the analysis of the predicted result in comparison to the true temperature value. Both are a measure of errors and both are independent of one another, therefore, it is possible to have a high measurement precision but less prediction accuracy.

For measurement precision test at a −2 dB SNR, it has been improved by 1.6 °C (1.71 MHz or 37.2 μϵ) in LM when using the double processing technique. This was followed by 1.7 °C, 0.7 °C and 0.5 °C improvement respectively for 3, 6 and 20 dB SNRs. It should be noted that there are two areas of the SNR-axis at Figure 8a showing stable differences of precisions between single and double processing—for SNRs below 2.6 dB, it is about 1.5 °C (1.6 MHz or 35 μϵ)—labelled as G1, and for SNRs above 7.6 dB, this value equals to 0.5 °C (500 kHz or 11.6 μϵ)—labelled as G2. This means that for SHM data with extremely low SNR, adding GLM after LM in the processing stage is necessary to keep the precision within acceptable level.

On the other hand, for BWC (see Figure 8b), the difference between single processing and double processing is around 0.4 °C (428 kHz or 9.3 μϵ), where single processing is the more precise technique for a −2 dB SNR. However, for other SNR conditions (above the SNR value of approximately 0.1 dB, which was found by approximation), the double processing technique is more precise than single processing. For 3, 6 and 20 dB SNRs, the difference is 0.3 °C, 0.4 °C and 0.3 °C, respectively. The measurement precision for double processing using BWC surpass the benchmark where it is 2.6 °C, 1.2 °C, 0.8 °C and 0.6 °C for −2, 3, 6, and 20 dB respectively. As for the double processing using LM, the precision is comparable to the benchmark.

A similar pattern can be seen in prediction accuracy plots. At a −2 dB SNR for LM (Figure 9a), the significant difference was found to be 8.3 °C (8.9 MHz or 193 μϵ). This was followed by 3.3 °C, 1.7 °C and 1.8 °C accuracy improvement for 3, 6, and 20 dB respectively. The graph shows that for SNRs below approximately 5 dB the “accuracy to SNR” slopes differ (marked as angles g1 and g2). It gives reason to suppose that further decrease of SNR could lead to the total signal loss when using a single processing technique with LM, while the application of double processing gives hopes for more precise BFS detection.

Comparably to the measurement precision, the single processing technique is more accurate at a −2 dB SNR for BWC (Figure 9b), where the difference was found to be 0.9 °C (963 kHz or 21 μϵ). The crossing of red and green lines occurs at approximately 0.2 dB, which is also found by approximation. However, a more accurate reading was scored by the double processing technique for other SNR conditions for BWC. The difference is 0.9 °C, 0.8 °C and 0.7 °C for 3, 6 and 20 dB SNRs respectively. The prediction accuracy for benchmark is found to be 4.3 °C, 1.9 °C, 1.3 °C, and 1.1 °C. This means that both double processing techniques scored a comparable accuracy result to the benchmark.

As can be seen from the figures presented above, double data processing is more efficient in almost all cases, both in the case of measurement precision and prediction accuracy. This suggests that the tasks set can be considered completed and we can proceed to a discussion of the general results obtained.

## 9. Conclusions and Future Work

In this work, we have successfully demonstrated the subsequent application of various methods for determining BFS, which gave a gain from tenths to several Celsius degrees when determining the temperature at a single point of an optical fibre. The graphs provided above showed that double processing is more efficient in most cases.

The conducted studies allowed us to conclude that the first part of the data processing algorithm should be finding the signal-to-noise ratio for each individual BGS. Depending on this value, the entire further signal processing strategy can be built. Thus, when processing signals with a low signal-to-noise ratio, it is recommended to use the BWC method as the first stage. With the SNR = 2 dB, even with single processing, it gives a gain of 0.5 °C (535 kHz or 11.6 μϵ) compared to the LM method. In the case of the BWC method, the double processing strategy should be as follows: at SNR <0 dB, more confidence should be given to the signal after single processing, and at higher signal-to-noise ratios, double processing is more effective.

The results obtained are in good agreement both with simulation and experiments in our previous works [40,41,46] and with the results of other authors. Thus, in [47], Haneef et al. examine a method called cross reference plot analysis (CRPA) and compare it with the well-known cross-correlation and LCF methods. The cross-correlation method, which is similar in nature to the backward correlation method, also exhibits higher accuracies in data regions with low SNR. The CRPA method itself, which also uses the convolution operation in its algorithm, gives the BFS determination accuracy at SNR = 6 dB about 1 MHz, which is slightly higher than that obtained by us. However, it is worth noting that for the CRPA method this accuracy is constant, while the artificial intelligence algorithm improves the accuracy with further training.

In [33], where the cross-correlation method for determining BFS is considered in detail, an accuracy of 2.7 MHz was achieved at a 12 dB signal-to-noise ratio, while our approach gives an accuracy of 0.7 MHz. Of course, it would be appropriate to carry out all such comparisons on the same initial data, but in fact, there are not many works where researchers compare their methods on different SNRs (even with different data). During the work on the material, we have not found a single scientific article that shows the fundamental possibility and successful application of combining BGS processing methods into chains (sequences), similar to how the methods of sequential processing of audio material are arranged in recording studios. We have proved that the formation of such sequences is possible due to the universality of the methods and the similarity of their input and output data. At this stage, double processing was the first step to multiprocessing.

We believe that for applications in SHM for concrete and reinforced concrete objects with normal operating conditions when the fibres are not subjected to extreme strains, the use of double processing is more appropriate. This strategy is suitable for increasing accuracy in most cases. According to [48], a fibre extension of 1000 μϵ (0.1%) results in an increase of only 0.108 dB/km in line attenuation. So when the fibre is stretched by units or tens of μϵ, the spectrum will not be dramatically distorted. A somewhat different situation becomes in the scientific problems of SHM of concrete structures. For example, when analyzing critical deformations of a reinforced concrete structure, much larger deformations (up to 2 mm/m, which is equivalent to 2000 μϵ or 0.2%) can be studied [49]. For a big research object such as a fragment of a residential building, extended lengths of optical fibre are used to obtain more information, then each kilometre with such deformations will reduce the SNR by more than 0.2 dB. Even more interesting is the situation with research on the shape memory materials [50]. In addition to the fact that they can have higher strains (up to 2.5%), they can also be below zero. This complicates the task and, provided that the integrity of the fibre is maintained, gives an almost twofold signal loss (>2.7 dB) per sensor kilometre. However, such a case is not frequent: usually, the dimensions of products made of shape memory materials are much smaller, so signal losses due to the impact on a long sensor are unlikely. At the same time, at fibre deformations close to breaking, when irreversible deformations are formed in the quartz glass and polymer coating, the dependence of the optical signal attenuation on the deformation magnitude can hardly be considered linear. In this regard, strong signal distortions are possible, thus the processing strategy in such cases must be studied in the future. The disadvantages of the proposed method include the obvious two-stage nature, which complicates the algorithm. However, this can be overcome by using more substantial computing resources. Their cost compared to the cost of BOTDA or BOTDR is extremely low. Another negative feature of the approach using a neural network is the requirement to train the system for each new application. However, it should be noted that any traditional method, when adapted to a new task, requires adjustment by a specialist, which also takes time; while the neural network learns on its own.

Another interesting area of future exploration may be the study of BFS in somewhat “exotic” fibres, for example, active ones. Such optical fibres have a complex multi-component structure—in addition to traditional silica and germanium oxides. They quite often contain aluminium oxide, and very rare earth elements such as erbium, ytterbium, holmium, etc. Each of these components significantly affects the final BFS, the accurate value of which is a “fingerprint” of a given sample and characterizes the correctness of the creation of its composition. However, such fibres have rather high optical losses (30 dB/m) in the region of 1.55 microns due to the presence of these elements. This makes the study of extended sections of such fibres by reflectometry methods rather difficult, although realizable [51,52,53,54]. In the case of Brillouin reflectometry, the use of short probe pulses leads to distortion of BGS, the appearance of a rather intense noise component. This task also opens a new page in the application of LM, BWC and GLM separately and together and can become a worthy part of research devoted to the multistage study of active fibres.

One more promising direction in the study of the sequential application of the LM, BWC and GLM methods can be their use in polarization-Brillouin reflectometry [46]. In such setup, linearly polarized radiation is injected into one or sequentially into different polarization axes using linear polarizers or polarization beam splitters. Each time light passes through such elements, it loses 3 dB of optical power or more. With the dynamic ranges of Brillouin reflectometers of tens of decibels and the presence of several forward and backward passages through such elements in the optical scheme, the study can be carried out with an extremely low signal-to-noise ratio. In some cases, the spectrum can show two superimposed Stokes or anti-Stokes of two polarization modes, which leads to a deviation of the shape of the resulting spectrum from the Lorentzian curve. Under these conditions, it would undoubtedly be interesting to evaluate the productivity of GLM, BWC and LM methods, which the authors propose to do in the future.

## Figures and Tables

**Table 1:** List of components and equipment used in the experiment.

No.	Components/Devices	Make and Model
1.	Laser source	Yokogawa AQ4312A
2.	Signal generator	Hittite HMC-T2220
3.	Pulse generator	Agilent 33521A
4.	Polarization scrambler	General Photonics PCD-104
5.	Mach–Zehnder modulator	iXblue MX-LN-20
6.	Single side-band modulator	iXblue MPX-LN-20
7.	Erbium-doped fiber amplifier	Keopsys CEFA-C-PB-LP
8.	O/E converter	Tektronix P6703B
9.	Oscilloscope	Teledyne-LeCroy HDO4054
10.	Optical spectrum analyzer	Yokogawa AQ6370B
11.	Fibre Bragg grating	Generic. FWHM 1nm. Reflectivity >95%
12.	Fibre under test	Corning SMF-28e+
13.	Polarization controller	Newport F-POL-APC
